# Supplementary material for: Sustained-input switches for transcription factors and microRNAs are central building blocks of eukaryotic gene circuits
Source: Genome Biol. 2013 Aug 23;14(8):R85. doi: 10.1186/gb-2013-14-8-r85 (PMC4054853; doi:10.1186/gb-2013-14-8-r85)
Supplement: Additional file 5 — HTML Browsable Motif Output. Zipped folder containing all WaRSwap and FANMOD motif output, viewable in a web browser. [file gb-2013-14-8-r85-S5.ZIP › HTML_browsable_motif_output/WaRSwap_all_tair9/sigs_WSWRSLDraw-2000_all.pvals.heatmaps.html/motif_id_166_010100111_tftype_all_upstream_-1000_0.html]

```
BG_MODEL = Rnew2
MOTIF_ID = 166_010100111
TF_TYPE = all
UPSTREAM = -1000_0


PVals
FN_0.2	FN_0.4	FN_0.6	FN_0.8
dg_60.genes	0.243	0.001	0.002	0.015
dg_70.genes	0.185	0	0.001	0.009
dg_80.genes	0.086	0.024	0.048	0.003

ZScores
FN_0.2	FN_0.4	FN_0.6	FN_0.8
dg_60.genes	0.159	6.295	6.051	-0.117
dg_70.genes	0.375	6.668	6.914	-0.096
dg_80.genes	0.746	1.731	-0.211	-0.055

StDevs
FN_0.2	FN_0.4	FN_0.6	FN_0.8
dg_60.genes	1.991	1.003	0.468	0.137
dg_70.genes	1.66	0.958	0.415	0.104
dg_80.genes	0.749	0.466	0.258	0.055
```
